# *Staphylococcus aureus* Infection: Pathogenesis and Antimicrobial Resistance

**DOI:** 10.3390/ijms24098182

**Published:** 2023-05-03

**Authors:** Giovanni Gherardi

**Affiliations:** 1 Clinical Laboratory Unit, Fondazione Policlinico Universitario Campus Bio-Medico, 00128 Rome, Italy; g.gherardi@unicampus.it; Tel.: +39-06-225411136; 2 Applied Microbiological Science Unit, Department of Medicine and Surgery, Università Campus Bio-Medico di Roma, Via Alvaro del Portillo 21, 00128 Rome, Italy

*Staphylococcus aureus*, a Gram-positive, coagulase-positive pathogen belonging to the family *Staphylococcaceae* with a spherical shape that forms grape-like clusters, is a commensal that is often present asymptomatically on parts of the human body [1]. *S. aureus* is also a major human pathogen able to adapt to diverse hosts and environmental conditions and cause many different infections. Additionally, it is one of the major causes of hospital and community-acquired infections. It can cause infections of the bloodstream, skin and soft tissues, and lower respiratory tract; infections related to medical instrumentation, such as central-line-associated bloodstream infection (CLABSI); and some serious deep-seated infections such as osteomyelitis and endocarditis [2,3,4,5]. *S. aureus* is equipped with a collection of virulence factors and toxins, the latter inducing numerous toxin-mediated diseases, including staphylococcal toxic shock syndrome, foodborne diseases, and scalded skin syndrome [6].

A major issue associated with *S. aureus* is its ability to acquire resistance to most antibiotics. Clinical use of methicillin has led to the emergence of methicillin-resistant *S. aureus* (MRSA), which is associated with high morbidity and mortality [7]. MRSA strains produce a new, altered penicillin-binding protein (PBP 2a or PBP 2′) associated with decreased affinity for penicillins, which is encoded by the acquired gene *mecA* carried on a mobile genetic element (MGE) named staphylococcal cassette chromosome mec (SCCmec) that can be acquired and inserted into the chromosomes of susceptible strains [8,9,10,11,12,13]. Another resistance determinant has been rarely identified among MRSA: *mecC*. MRSA isolates are typically resistant to all available penicillins and most other beta-lactam drugs except ceftaroline and ceftobiprole. Vancomycin has historically been the drug of choice, and it is sometimes considered the last line of treatment for severe MRSA infections. However, vancomycin is considered less effective than penicillin, and its increased use has been associated with the rise of vancomycin-intermediate *S. aureus* (VISA) and vancomycin-resistant *S. aureus* (VRSA) in some regions. Importantly, rapid detection of MRSA infections, mainly bloodstream infections, via phenotypic and genotypic methods and early communication of results in conjunction with antimicrobial stewardship can potentially improve care, facilitating reductions in unneeded antimicrobial use, antimicrobial resistance, and costs [14].

Previously, MRSA was primarily associated with healthcare settings (the so-called hospital-associated MRSA (HA-MRSA)). However, community-acquired MRSA (CA-MRSA) infections have been increasing and currently represent a major cause of community-associated infections [15]. CA-MRSA isolates are genetically different from HA-MRSA; for instance, CA-MRSA are resistant to fewer non-beta-lactam antibiotics, carry a smaller trait of SCCmec, and often produce Panton–Valentine leucocidin [16]. CA-MRSA has been reported to invade healthcare settings and thus cause nosocomial outbreaks [17]. Beside humans, MRSA colonization and infection have also been reported in animals, for example, in livestock, companion animals, and wild species [18]. The abuse and misuse of antimicrobial agents in these settings has strongly contributed to the spread of MRSA among livestock [6]. Numerous studies have described colonization and infections caused by livestock-associated MRSA (LA-MRSA) in humans in contact with livestock. Thus, livestock and other animals may represent an important permanent reservoir for human MRSA infections [6]. Due to the significant public health problem it has generated and the limited available treatments for MRSA infection, new, recently approved antibiotics with antibacterial activity, such as telavancin, dalbavancin, oritavancin, and tedizolid with potent in vitro activity against MRSA isolates, have been described, thereby highlighting a future direction for the introduction of useful antibiotics for treating MRSA infections [19].

In this Special Issue, original research articles that discuss the pathogenesis and novel therapeutic approaches for treating *S. aureus* infections in both humans and animals have been collected. Mayer et al. [20] uncovered an association with within-host adaptation, a typical feature of chronic, persistent *S. aureus* infections, in a bovine mastitis infection with increased cytotoxicity. Adaptive processes of *S. aureus* during chronic, persistent bovine mastitis were investigated in an isolate from a dairy cow with chronic, subclinical mastitis using a combinatory approach of surfaceomics, molecular spectroscopic fingerprinting, and in vitro phenotypic assays. The authors concluded that the within-host-evolved SigB-deficiency strain variant might favor extracellular persistence in *S. aureus* infections.

*S. aureus* infects epithelial cells, but the interaction between *S. aureus* and its host has not been adequately explained. Yang et al. [21] elucidate a new aspect of the mechanisms of infection and immune system evasion for *S. aureus*. They describe the ability of *S. aureus* to be internalized by HaCaT cells using the protein EsxB by escaping host immunity. *S. aureus* was found to increase the expression of decay-accelerating factor (CD55) on the surfaces of host cells, which inhibits the activation of the complement system and facilitates survival in host cells, and the infected host cells increase their surface expression of UL16 binding protein.

The other three papers conducted studies on methods for combatting the emergence of antibiotic resistance in *S. aureus* by proposing novel antibacterial strategies. Chu et al. [22] investigated and characterized the nusbiarylin compound MC4 and several of its chemical derivatives in both MRSA and the *S. aureus*-type strains. They provided evidence of these compounds in the capacity to inhibit, on the one hand, growth, cellular respiration, and transcription and, on the other hand, attenuate virulence factors, such as the exoproteins α-toxin and Panton–Valentine Leukocidin, possibly by acting via the modulation of global regulatory pathways. Lee et al. [23] investigated the antibacterial activity of the HP (2–20) peptide, which was derived from Helicobacter pylori ribosomal protein L1 and modified with d-Lys residues, against *Escherichia coli* and *S. aureus* of an analogue. This peptide showed excellent antimicrobial activity and no evidence of any induction of resistance. Finally, the “in vitro” and “in vivo” antibacterial effect of a novel ruthenium-based coordinate compound was investigated by Sur et al. [24] against one *Staphylococcus epidermidis* isolate and three *S. aureus*-type strains with different antibiotic resistance patterns: one susceptible, one vancomycin-resistant, and one methicillin-resistant (MRSA). All the infected mice were cured, and the compound resulted in being non-toxic toward mammalian cells and those of mice.

This Special Issue provides new insights into mechanisms of pathogenesis and proposes novel potential antibacterial drugs for use as potent antimicrobial agents for combatting *S. aureus* infections.
